# A Dual Perspective of Psycho-Social Barriers and Challenges Experienced by Drug-Resistant TB Patients and Their Caregivers through the Course of Diagnosis and Treatment: Findings from a Qualitative Study in Bengaluru and Hyderabad Districts of South India

**DOI:** 10.3390/antibiotics11111586

**Published:** 2022-11-10

**Authors:** Karikalan Nagarajan, Karthikeyan Kumarsamy, Rehana Begum, Vikas Panibatla, Rameshchandra Reddy, Rajesham Adepu, Joseph Francis Munjattu, Senthil Sellapan, Stephen Arangba, Amrita Goswami, Reuben Swamickan, Malaisamy Muniyandi

**Affiliations:** 1 ICMR-National Institute for Research in Tuberculosis (NIRT), Chennai 600031, India; 2 Karnataka Health Promotion Trust, IT Park, Rajajinagar Industrial Area, Bengaluru 560044, India; 3 TB Alert India, Hyderabad, West Marredpally, Secunderabad 500026, India; 4 State TB Office, Lady Willingdon State TB Centre, Bengaluru 560027, India; 5 State TB Office, Directorate of Medical & Health Services, Hyderabad 500095, India; 6 ICMR-RMRC Port Blair Andaman, Nicobar Islands 744103, India; 7 Infectious Disease Division, New Delhi 110021, India

**Keywords:** drug-resistant tuberculosis, caregivers, emotional distress, hopelessness, fear, decisive moments, pill burden, nutrition, pain, injections

## Abstract

Qualitative insights regarding psycho-social barriers and challenges experienced by drug-resistant tuberculosis (DR-TB) patients and their caregivers are understudied in India. We conducted a qualitative study using semi-structured qualitative interviews among treatment-completed DR-TB patients (*n* = 20) and caregivers (*n* = 20) in Bengaluru and Hyderabad districts, which represented two different socio-cultural settings in South India. Criterion sampling was used for recruiting the eligible participants who completed treatment with adherence. “Emotional issues and social barriers” were identified to represent a major challenge for patients and caregivers, which occurred acutely after disease diagnosis, characterized by fear and emotional distress due to their perceived loss of life prospects, severity of symptoms, discomfort, and disease denial. Medication intolerance, chronic symptoms, lack of visible signs of treatment progress, loss of weight, and physical concerns caused subsequent fear and distress during the treatment phases for patients along with experiences of stigma. External triggers generated “decisive moments” of hopelessness and life-ending thoughts for patients at the diagnosis and early treatment phase. Medication related challenges included the perceived burden and power of pills which caused emotional distress for patients and intolerance towards caregivers. Pill burden was found as consequential as the side effects of injections. Challenges related to lack of support were another major theme, in which caregivers lacked resources for treatment support and nutrition. Throughout treatment, caregivers and patients expressed concern about a lack of supportive care from family members, sympathy, and intangible social support. Challenges during hospital admission in terms of lack of privacy, quality of services, individual attention, and empathy from health care workers were reported by patients and caregivers. Despite better adherence, DR-TB patients and caregivers experienced considerable emotional and social consequences. Differentiating DR-TB patients and caregivers’ issues at different stages of diagnosis and treatment could help improve patient-centered outcomes in India and other high-burden nations.

## 1. Introduction

Drug-resistant tuberculosis (DR-TB) remains a grave public health threat globally with an estimated 4.1% of new cases and 19% of previously treated cases having some form of drug resistance. India is one of the countries with the largest number of DR-TB patients accounting for one-third of the global burden [1]. The first national drug-resistance survey in India showed that resistance rates among newly diagnosed TB patients was 2.84 percent and 11.60 percent among previously treated TB cases [2]. As of 2020, a total of 66,359 DR-TB cases were recorded, and 56,500 (85%) of them were put on treatment in India [3]. In 2019, DR-TB patients treated with newer, shorter regimens had a moderate treatment success rate (60%), and higher fatality rate (11%), and higher loss to follow-up rate (13%). Despite the introduction of newer, shorter regimens, treating DR-TB patients is still complex and challenging [3]. Treatment outcome patterns among DR-TB patients have been noted with significant rates of loss to follow-up, mortality, and treatment failure in India and other countries [4].

DR-TB is caused by mycobacterium tuberculosis strains which are resistant to first -line medicines like isoniazid and rifampicin that are more potent in treating TB. DR-TB patients require treatment regimens which are longer and have higher possibilities for adverse events. DR-TB treatment includes longer regimen of 20 months and shorter regimens of 9–12 months which include a combination of oral drugs and/or injections. The adverse psycho-social effects of DR-TB treatment include depression, irritability, stress, altered consciousness, suicidal tendencies, neuro psychiatric effects, and poor quality of life. The relationship between psycho-social problems and treatment adherence and the outcome of DR-TB patients are bidirectional in nature. DR-TB patients experience adverse biological debilitation and rapid progression of the disease, which itself is a reason for mental agony, hopelessness and poor uptake of treatment. Alternatively, the treatment complexity of DR-TB and its associated toxicity contributes to mental distress and psycho-social problems. A wide range of biological side effects of DR-TB drugs includes anorexia, vomiting, injection-induced pain, drug-induced psychosis, severe gastro-intestinal disturbances, arthralgia, hepatotoxicity, neuropathy, ototoxicity, and nephron toxicity, which creates mental distress and agony. Thus, the treatment outcomes of DR-TB patients are affected by the vicious cycle of disease severity, treatment complexity and psycho-social challenges which mutually reinforce each other.

Irrespective of regimen types, DR-TB treatment continues to present challenges to patients, caregivers and health systems due to the length and side-effects of these regimens, which increase the morbidity and mortality. In addition, stigma, discrimination, out-of-pocket expenses, catastrophic expenses, and poor quality of life are the major difficulties which the DR-TB patients’ experience during treatment [4,5]. While developing less invasive and shorter duration regimens for DR-TB treatment could lead to decreased complexity and increased treatment adherence and outcomes, equal importance is required to understand and address the psycho-social and economic problems faced by DR-TB patients.

Research in India on the treatment-related psychosocial challenges and barriers faced by DR-TB patients are scarce and very few studies have used qualitative methodologies for in-depth exploration of patient experiences and perspectives [5,6,7,8,9]. The adaptation of rigorous qualitative methodologies with appropriate representation of stakeholders and socio-cultural representation of participants remains a gap in India. We aimed to incorporate both the DR-TB patients and their primary caregivers in a comparative way, from two different socio-cultural settings, to add to the current evidence and to broaden the insights on the challenges and barriers faced by them in India. In a society where family care system holds a predominant role in caring for unwell people, it is critical to understand and compare the patients’ hurdles and difficulties from the perspective of the caregivers which had not been attempted before.

In India, family caregivers who share considerable social and psychological problems along with DR-TB patients have received less research attention [10,11]. While HIV research in India had looked at the perspectives and experiences of caregivers, still, in the context of DR-TB, which is comparably severe and where family care is critical to a patient’s adherence to treatment, such a perspective is still lacking [12]. Second, our research intended to identify psycho-social problems and barriers concerning the patients journey from the point of diagnosis to treatment completion, to acquire a timeline perspective. Previous qualitative research has not attempted to distinguish the dynamic barriers based on the diagnostic and treatment phases of the DR-TB patients [13]. To add to this perspective, we attempted to analyze and identify barriers and challenges reported by DR-TB patients at three distinct stages of their diagnostic and treatment path, similar to what has been attempted among HIV/DR-TB patients in South Africa [14]. This study also attempted to explore whether treatment adherence challenges remain hard for DR-TB patients who finished treatment with better adherence, as observed for less adherent individuals. In this context, the present research aimed to explore, differentiate, and comprehend the treatment-related difficulties, psycho-social barriers, and challenges faced by more adherent and treatment completed DR-TB patients and their primary family caregivers in Bengaluru and Hyderabad districts, which represented two different socio-cultural geographies of South India.

## 2. Materials and Methods

### 2.1. Study Settings

This qualitative study was conducted between 2020–2022 in the Hyderabad district of Telangana state and the Bengaluru district of Karnataka State of South India. Telangana is an agriculturally and economically developed Southern Indian state with a population of 35 million and a high literacy rate (>65%). The capital district Hyderabad is a developed metropolitan area of the country with a population of 4 million. In 2019, a total of 52,139 TB cases were notified in Telangana state of which 1507 were DR-TB cases. Similarly, Karnataka is another southern state with a high population (60 million), high literacy rate (75%) and development indices which is situated adjacent to Telangana state. Its capital district Bengaluru is one of the important industrial cities in the world with a population of 8 million. In 2019, a total of 81,388 TB cases were recorded in Karnataka state of which 1696 were DR-TB cases.

### 2.2. Study Population

Criterion sampling was used for this study in which participants were selected based on a pre-defined criterion. In this type of sampling the criteria that are important to the research are pre-determined and this necessitates the researcher to identify and study the participants who meet them. Participants are selected on the basis that they have knowledge and experience with the phenomenon of interest and therefore will be able to provide information which is in-depth [15].

In the present study, we used criterion sampling to identify positively deviant adult (>18 years) DR-TB patients who had successfully completed DR-TB treatment without interruption for ≥2 consecutive days. Positive deviants referred to individuals who stand out from their peers in terms of dealing with the challenges they face in common and have better outcome than their peers [16].

### 2.3. Ethical Approval and Human Rights

The Institutional Ethics Committee of the ICMR-NIRT in Chennai and the Institutional Ethics Review Board of St. Johns Medical College in Bengaluru both approved the study. Participants were given a participant information sheet, with which the study was thoroughly explained to them, and written consent was obtained from them.

### 2.4. Data Collection

DR-TB patients who underwent treatment under the public sector health facilities and enrolled in the National TB Elimination Program (NTEP) of the study districts were used as the sample population of this study. A positive deviance approach was used to identify the subset of DR-TB patients with better adherence. The treatment information of the participants for the study period was retrieved from “NIKSHAY”, a web enabled patient management system for TB control under NTEP of India. NIKSHAY is used by health care providers, administrators, program managers, and policy makers of TB at various levels across the country both in the public and private sector, to register cases under their care, record treatment details, monitor treatment adherence, refer for testing, and to transfer cases between care providers based on needs [17].

Further, the study employed a multi-step procedure to identify the eligible participants. Frontline health care providers (HCP), who were supervising the treatment of patients were inquired about the treatment regularity and completion status. The study team comprised both of male and female, qualified and trained social workers and public health researchers who inquired with patients and assessed their criteria for study based on their treatment cards. After the eligible participants were identified, a detailed participant information sheet was provided and were explained about the study objectives specifically and subsequently informed consent was administered. Further, each one was asked to nominate a primary caregiver who had enabled him or her in completing the treatment. Face-to-face, semi-structured interviews (SSIs) were utilized to investigate, identify, and comprehend the psychological, social, and behavioral barriers and challenges that patients faced. Caregivers were interviewed to get their perspectives on their patients and also their own experiences.

A total of 252 DR-TB cases were line listed from the NIKSHAY in Bengaluru, of which 192 were shortlisted by HCPs as patients who completed their treatment with the highest level of adherence. By cross-checking, the study team identified 78 patients (of *n* = 252) as suitable for the interviews as per the study criteria. Similarly, in Hyderabad, 116 DR-TB cases were line listed, of which health workers selected 73 patients as better adherent patients. Further, the study team narrowed it down to 36 cases (of *n* = 116) as per criteria. Eligible patients in both sites (*n* = 114) were approached and after excluding for language barrier, non-willingness for audio taping, and consent (*n* = 28) and guided by principles of saturation, a total of 20 patients and their caregivers (*n* = 20) were recruited in both sites. The saturation principle underscored that no additional qualitative data are required to interpret the data and develop thematic constructs.

### 2.5. Study Process

A semi-structured interview guide with probes was used to explore the patients’ physical health, mental well-being, fear and hopelessness, social life, work, education, income, and financial situation with reference to their diagnosis, treatment initiation and treatment completion phases. Participants were asked to discuss their TB diagnosis and treatment experiences, as well as the obstacles and barriers they faced. Concerning treatment-related challenges and their mediated impacts, specific probes were used to understand their financial and occupational issues, lack of support, lack of information, stigma, and discrimination issues. The family caregivers were asked about the same experiences of the patients from their perspective, as well as their barriers and challenges in their caring giving role. The semi-structured interviews were conducted by certified social workers and public health researchers who had received significant training in qualitative interviewing. The interviewers were not involved in patient care or adherence in the same hospital. The phrasing and sequencing of probes and questions were changed to fit the patients’ response pattern and dynamics based on a pilot interview with two participants in each site. Whenever there was a lack of sufficient information from participants, specific probes were refined after discussions with the data collectors. Probes related to health care providers, intimate relationships, or traumatizing experiences of stigma and discrimination were mostly rephrased. The caregiver of the patient was also interviewed using the same method but with interview guides specific for them. Caregivers shared their experiences and perspectives about patients challenges and also shared their own experiences in the care giving process. All interviews took place in their local languages (Kannada, Tamil, Telugu, Urdu and Hindi). Each interview lasted for approximately 45–60 min.

### 2.6. Data Analysis

The analysis was conducted based on a systematic and multi-step process involving familiarization with raw data, generating coding framework, checking saturation, identifying, reviewing and organizing sub-themes and major themes. Initially interviews were taped, and further notes were transcribed from local language to English. The transcribing quality was evaluated, and the translation was performed in English. Linguistic nuances were considered, and the quality of the translation was ensured. Iterative preliminary analysis of interviews by (R.B., V.P., K.N.) resulted in the identification of preliminary coding framework. The transcript files were imported into NVivo 12 (QSR International) software for in-depth thematic analysis [18,19,20,21]. Top level codes were developed which provided clarity and distinguishable patterns of participant perspectives and experiences. Further sub-themes were developed based on the similarity of top-level codes, and were further categorized into major themes. The analysis team comprising of two members (K.N., M.M.) iteratively evaluated the emerging top codes and sub-themes throughout the analysis and arrived at the final set of themes at the end of the analysis based on the saturation which was attained at the 19th patient interview. Quotes and analytical memos were reviewed and placed under the appropriate thematic heads. Consensus was arrived at wherever there was disagreement on themes and sub-themes categorization. Participants were reached out whenever there is a need for additional clarity of quotes to help arrive at a consensus. An additional code was added to the analysis to help categorize some of the sub-themes of the patients’ diagnostic and treatment phases. Thus, a timeline approach was used to describe sub-themes wherever possible. Caregivers’ interviews were analyzed independently initially, and the themes were found to be consistent with those of patients. Patients’ and caregivers’ themes and sub-themes were coded jointly and differentiated using a framework matrix during analysis. The perspectives and experiences of patients and caregivers were compared to see what they have in common. The study report was in confirmation with the CONSORT guidelines (Supplementary File S1).

## 3. Results

### 3.1. Demography

Twenty DR-TB patients and their caregivers were interviewed for this study. The average age of the DR-TB patients was 36 years and the proportion of male patients was higher (65%, *n* = 13). The average age of caregivers was 42 years and the proportion of female caregivers was high (70%, *n* = 14). All caregivers were family members. In terms of literacy, two-thirds (>70%) of male and female patients were school-educated and almost one-fifth of the caregivers were illiterate (21–33%). In terms of treatment, all patients underwent shorter treatment regimen for DR-TB with injections. (Table 1).

#### Main Themes and Sub-Themes Classification

**Figure 1:**
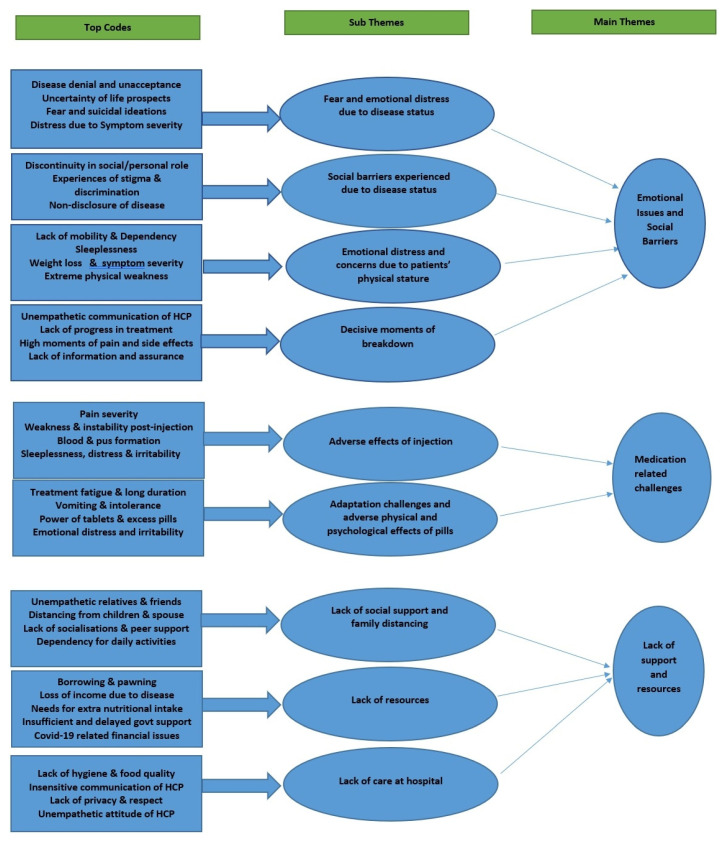
Hierarchical representation of the emergence of codes, sub-themes and main themes.

Three distinct themes emerged concerning the challenges and barriers faced by DR-TB patients and their caregivers in continuing treatment: (1) emotional issues and social barriers faced by patients and their caregivers, (2) medication related challenges and barriers, and (3) lack of resources and support reflected the challenges and barriers in terms of economic and social support access for the patients and caregivers. For each of these major themes, distinct sub-themes were identified. All of the sub-themes that emerged had numerous identifiable patterns indicating the challenges faced by the patients and caregivers from the point of diagnosis through the end of treatment. Overall, there were patterns observed in which the responses of the patients and their family members and caretakers were comparable, emphasizing the oneness of their experiences, which are detailed in the following sections (Figure 1 and Supplementary File S2).

### 3.2. Main Theme-1: Emotional Issues and Social Barriers (Table 2)

#### 3.2.1. Sub-Theme-1: Fear and Emotional Distress Due to Disease Status

At the time of diagnosis and treatment, the patients’ fear and emotional distress were attributed to their own doubts about life prospects and skepticism about the return to normalcy. Following diagnosis, patients perceived a loss of personal prospects, uncertainty about the future, concerns about survival, concerns about the severity of symptoms, sense of righteousness and moral hurt over the diseased condition, and disease denial. This subtheme showed distinguishable patterns dependent on the patients’ diagnosis and treatment phases. Patients and caregivers were both worried and distressed about their children’s future and life prospects. Fear was more attributed to their personal and family members’ future status rather than the disease situation. Patients and caregivers voiced their distress at the time of diagnosis, wondering why TB had re-infected them after living a healthy lifestyle. They felt it was unfair that they were infected despite following proper health-seeking measures and being morally good. The distress was expressed in the form of self-questioning their morality and self. Furthermore, patients and caregivers were much more worried at the time of diagnosis about the severity of disease symptoms and their worsening physical appearance, which was unfathomable to them, and they voiced their pessimism over their declining physical state.

Fear and emotional distress reported by patients during the early phase of treatment were distinct from those encountered during the diagnosis period. The intake of drugs with severe side effects of the treatment stage worsened the already existing emotional distress of the patients caused by the severity of symptoms. The impact of this entire situation was not only experienced by the patients, but it had an impact on caregivers too. The caregiver’s confusion and cluelessness even after long months of treatment was another reason for the fear and emotional distress experienced by them.

#### 3.2.2. Sub-Theme-2: Social Barriers Experienced Due to Disease Status

Another sub-theme that emerged significantly during the diagnosis and treatment stage of DR-TB patients was the social challenges faced by the patients and their caregivers. Following the diagnosis, patients expressed that they have detached from society and have stopped playing an active social role. They were unable to pursue their studies or work due to financial constraints. Fear of being identified by their symptoms, loss of interest, and challenges caused by TB were cited as the reasons for leaving their jobs or schools. Patients experiencing difficulties adjusting to society during the early and late stages of their treatment were alike. At this stage of the treatment journey, enacted stigma and discrimination had emerged as the key theme defining social relations.

#### 3.2.3. Sub-Theme-3: Emotional Distress and Concerns Due to Patients’ Physical Stature

Both the patients and the caregivers expressed emotional distress and concerns due to the patients’ physical stature. The patients voiced concern over losing weight, physical inability, and consequent dependence, which had a psychological and emotional impact on them. From the time of diagnosis through the end of treatment, this pattern remained consistent.

#### 3.2.4. Sub-Theme-4: Decisive Moments of Breakdown

Many patients described an acute phase of experience or event which brought them to the point of giving up hope of life and treatment during post-diagnosis and early stages of treatment. Such decisive moments were triggered by the patients’ external circumstances, resulting in a comparable situation of hopelessness and a sense of defeat for them.

### 3.3. Main Theme-2: Medication-Related Challenges (Table 3)

#### 3.3.1. Sub-Theme-1: Adverse Effects of Injection

The experiences with the injection were very consequential for the patients and their families. Injection experiences were identified with unbearable pain, swelling, discomfort, blood, pus, fluids, sleeplessness, giddiness and an array of other issues. Both the patients and family members expressed the challenges of taking injection during the treatment. The pain of injection was considered the top reason for despair for both patients and their families. Injection-induced disability was cited as a major concern by few patients.

#### 3.3.2. Sub-Theme-2: Adaptation Challenges and Adverse Physical and Psychological Effects of Pills

During the early phases of treatment vomiting, nausea, burning sensation, dizziness and a wider range of issues were cited as the most common challenge for all the patients that occurred immediately after consuming the tables. The caregivers also narrated that the side effects and the “power” of medicine were intolerable for the patients to assimilate. The high dosage was linked to the high power of medicine which they perceived to have caused physical weakness to them.

Pill burden and medication adherence were expressed as the major challenge by the patients and their family members during the later phase of treatment. The large number of tablets which the patients have to take and the long duration of the treatment which they have been instructed to follow was the key source of frustration, anger and helplessness for both the patients and the caregivers. The patients and the caregivers narrated that the challenges pertaining to number of pills to consume and the lengthy duration of the treatment were equal to that of the side-effects of injections.

### 3.4. Main Theme-3: Lack of Support and Resources (Table 4)

#### 3.4.1. Sub-Theme-1: Lack of Social Support and Family Distancing

The denial or absence of any form of help from expected relatives and other social members was perceived by the patients and caretakers as very negative and sorrowful during their treatment time. It was underscored by the patients and their families as an important factor which bothered them a lot. The need for supportive care from the family members during the treatment was expressed by the patients owing to their inability to take care of themselves during the initial months of treatment.

#### 3.4.2. Sub-Theme-2: Lack of Resources

The lack of resources to cope with the treatment was highlighted by the patients and family members. Loss of income, food insecurity and expenses for the treatment was highlighted during the treatment period. The lack of support from close social networks was underscored in terms of the money needed to cope with the treatment. Many patients also positively recognized the monetary support provided by the government for TB treatment, but also underscored the insufficiency of the amount to meet their daily needs and delays or gaps in receiving the money. The mitigation of resource needs was mostly through personal strivings and outreach. Patients and caregivers expressed that extra and special nutritional needs to overcome the disease were the main reason for the economic burden. Patients expressed that the provision of monthly financial support given by the NTEP program was useful but was not sufficient and not delivered on time to meet the immediate and critical needs of them and their families during the treatment period.

#### 3.4.3. Sub-Theme-3: Lack of Care at the Hospital

Most patient expressed that the care provided to them during the initial admission period was uncomfortable due to the lack of positive attitude, lack of respect for privacy, as well as a lack of empathy and individual attention towards their difficulties from the health care providers. The hospital environment and poor hygiene was a factor of concern for the patients and caregivers. However, there was also an understanding that the initial treatment days at the hospital were important and useful despite of the concerns they had.

## 4. Discussion

The findings of this study provide an in-depth and critical understanding of the psycho-social barriers and challenges that DR-TB patients and their caregivers confront. The study findings and the new conclusions have practical significance for the DR-TB patients and NTEP in India. First, for attempting to understand the psychological and social issues of the patients together with the perspective of caregivers. Second, for attempting to align the differential challenges and barriers experienced by DR-TB patients alongside their treatment journey. In this context, the current study found that the fear and emotional distress that patients and caregivers perceived and experienced varied dynamically and emerged as the most defining theme.

Patients and caregivers expressed concerns and fears about the disease impact on their own and family members lives and future prospects. This underscored the unmet need for a timely reassurance and inculcation of a belief in patients and caregivers about the reversibility of sickness and return of normalization. It has been shown in previous research that, in addition to the disease-related effects of DR-TB, the social consequences of the disease are equally crucial. The disruption that DR-TB causes in an individual’s ability to fulfil social and familial responsibilities were particularly worrisome and frightening for patients which needs timely intervention at the time of disclosing the diagnosis [5,22]. The disclosure process of DR-TB diagnosis could be made in a more sensitive and guided way as similar to other disease conditions, such as HIV and cancer, which could prepare the patients and caregivers mentally and socially [23,24].

Patients exhibited extreme fear and helplessness as a result of symptoms that were inexplicable and unfathomable to them. Patients and caregivers were found to be in denial about disease after diagnosis, which could be attributed to the low self-risk perception and lack of understanding about the etiology of DR-TB [25]. This refusal to acknowledge the disease status might be explained in the context of underlying cultural belief system, which associates tuberculosis with negative deeds of individuals [26]. Denial to accept the disease condition requires intervention which could help unlearn the patients about the myths and wrong beliefs about the causes of TB. Interventions which could facilitate experience sharing from other patients through support groups could be of use to address disease denial. Moreover, providing basic TB literacy to diagnosed patients and caregivers in an early stage could address such moral issues and educate them on the severity of expected symptoms and possibilities of recovery [27].

Persistence of symptoms and the perceived ineffectiveness of medications was the causative factor for additional emotional intolerance as the treatment progressed. At this point, the caregivers were also exposed to the patients anger and intolerance [28]. Such later-stage experiences would necessitate consistent sensitization of patients about their treatment progress based on positive feedbacks and helping them to avoid misperceptions and self-judgments based on unpleasant symptoms [29].

### 4.1. Acute Phases of Hopelessness and the Need for a Special Approach

Patients experienced certain “decisive moments” triggered by some unpleasant events during the early stages of treatment that culminated in a feeling of hopelessness and quitting of medications. While conventional counselling and reassurance aim to treat the patients’ psychosocial issues in a systematic manner, such unanticipated emotional triggers and decisive moments occurring for patients highlight the importance of crisis response. Lessons from suicide prevention, cancer care, and HIV/AIDS programs offer examples for establishing a crisis response for patients in acute need of redressal and care which could be adopted in DR-TB context [30,31].

### 4.2. Lack of Supportive Care and Social Support for Patients and Caregivers

The gender profile of caregivers highlights the importance of gender imbalance when it comes to caring for DR-TB patients in families and also the burden of responsibility and shared emotional distress. The importance of caregivers of DR-TB patients in India which shares one fourth of the global burden of DR-TB is less studied and lacks recognition [32,33]. Creating caregiver networks and enabling support groups among caregivers could be of use for them to cope better [34].

### 4.3. Physical Appearance Driven Anxiety and Distress

Patients’ attribution of drastic weight loss, physical weakness, and immobility to poorer treatment effects appear to have a negative influence on their mental well-being. Such circumstances highlight the importance of reinforcing the communication of positive information about the patients’ health progress on a regular basis by the health providers during treatment visits [35].

### 4.4. Lack of Resources as a Major Barrier

While the financial burden of DR-TB patients has been reported previously, the influence it had on the family’s financial prospects, as well as the central role of family members in dealing with the condition is uncovered from the present study. While the DR-TB treatment, including hospitalization charges, are provided free of cost by NTEP, the indirect costs associated with treatment (incurred for food, travel, loss of wages, time lost, extra livelihood needs, injection access cost) and for the caregivers were still noted to be significantly higher. Even after a decade of research into the devastating economic consequences of DR-TB, there are currently no appropriate systems or policies in place to address the issues. Provision of insurance coverage for DR-TB patients and caregivers to meet their catastrophic expenditures could be of use if implemented efficiently in India. [9,36,37,38].

### 4.5. Adverse Physical and Psychological effects of DR-TB Injections and Pills

While the current study participants have finished short-course DR-TB treatment with favorable treatment outcomes and maximum adherence, their overall experiences with injections and tablets remain mostly unfavorable and consequential, comparable to those of poor adherents of DR-TB medications [5,39]. The experience with high dosages of tablets resulted in psychological exhaustion and discomfort which lasted longer for patients [5]. While the side-effects of injection for DR-TB patients may be mitigated in the near future with all oral regimens, still tablet consumption may continue to be a substantial barrier and source of mental distress for DR-TB patients [5]. These findings compel us to focus on possible approaches, e.g., pill planning and patient friendly prescription strategies, to mitigate pill burden related issues [40,41]. The intolerance and distress shared by caregivers and their key role in patient outcomes has been understudied in the context of DR-TB care in India [5,41,42,43]. While culturally, caregivers are more accepting and understanding of patients’ problems, enduring emotional distress while caring the patient for an extended period of time could still have negative consequences for the caregivers themselves. The dual-impact of DR-TB treatment on patients and caregivers must be given equal importance and underscores the need for developing interventions to ameliorate such impacts [44].

Our study findings need to be interpreted with some limits. Our study sample included adult DR-TB patients and their caregivers from public health facilities. However, the perspectives of DR-TB patients who are children and adolescents, and patients who did not have caregivers might be different from that of our study population. Future studies among such sub-populations of DR-TB could be undertaken.

In summary, the current study adds to our understanding of the treatment-related and psychosocial issues faced by DR-TB patients and their caregivers across two socio-cultural geographies in India. The current study explored and added the perspectives of caregivers of DR-TB patients, which has not been done before in India. The findings highlight the shared burden of treatment and psycho-social challenges by the caregivers of DR-TB patients. The study also identified a dynamism in few aspects of psycho-social issues confronted by DR-TB patients along their treatment journey. which has not been attempted in India [14]. Another key finding of this study is that it highlights the enormous psychosocial challenges and problems that the DR-TB patients and caregivers endure, despite having completed treatment with better adherence. Documenting such challenges and constraints of DR-TB patients from a broader perspective over and above the treatment adherence concerns and developing relevant interventions to comprehensively address them is critical for improving overall DR-TB patient outcomes in India and other high-burden countries.

## Figures and Tables

**Table 1:** Demographic details of participants.

Category	Characteristics	No	Average Age	Literacy		
				School	Illiterate	Graduate
Patients	Male	13	38	10	1	2
	Female	7	34	5	1	1
Contacts	Male	6	35	3	2	1
	Female	14	50	8	3	3

**Table 2:** Quotes reflective of the theme “Emotional issues and social barriers” and its sub-themes.

3.2. Main Theme-1 Emotional Issues and Social Barriers	
3.2.1. Sub-theme-1: Fear and emotional distress due to disease status	
“When I was diagnosed with TB disease, I was extremely worried and fearful about my condition. I was worried and skeptical about overcoming the disease. I introspected and feared thinking, Will I be able to get through this (TB)? Will I be able to see my child once again?”	Patient (Male, aged 33 years) (Code B1)
“I am scared. We are going to give her to others (in terms of marriage). I wished her a speedy recovery from it (TB), as I was worried about her future. I was worried that if she could get married or not. I was scared that it might be a problem later in her life”	Caregiver (Female, 54 years, Housewife) (Code B10F)
**3.2.2. Sub-theme-2: Social barriers experienced due to disease status**	
“No. I left the job soon after I was diagnosed with (TB). I was not going to work or outside because I had a severe cough and I was feeling weak and tired. Whenever I was coughing in front of them, they used to sarcastically and rudely tell, what is wrong with you? Why are you coughing so much? If you have health issues go to the hospital, don’t come here”	A patient (Male, 28 years) told (Code B5)
“They (people) were discussing amongst themselves; he is the pastor and his wife is suffering from this kind of disease. He has his own church and prays for so many people, but why this thing has happened to him and his family? That kind of word was hurting me and was painful”.	A female caregiver (30 years) (Code B2F)
**3.2.3. Sub-theme-3: Emotional distress and concerns due to patients’ physical stature**	
“I lost weight a lot and turned pale. I was not able to go to work, I had no appetite. I use to sleep most of the time. I became very weak and lost interest in work. I lost weight from 40 to 30 kg and then to 28 kgs. During the initial stage itself, I was vomiting blood”	Patient (Female, 25 years) (Code B3)
“For six months, I had struggled a lot to take care of him. Then as it went on, we got used to it. Initially, for one month, he was like a bed patient, he used to feel tired and weak and always lying on the bed”	Caregiver (Female, 47 years, wife) (Code B9F)
**3.2.4. Sub-theme-4-: Decisive moments of breakdown**	
“When I asked the reason, he told, I can only survive up to only 40 years. Your validity is 40 or 45 plus because of your disease condition. I went there with higher hopes but he pulled me down with no hopes left inside. I lost my mind there only…. And soon after reaching home, I decided to quit my life”	Patient (Male, 28 years) (Code B5)
“At that time after taking the injection, there will be a lot of difficulties sir. For two months I have to take injections daily even though it was on Sundays or holidays. For ten days I took an injection and stopped one day, again they said the injection will not work if I stopped for a day. They told me that again I have to take back all the ten days of injection. At that time my health was very weak so I lost my hope”.	Patient (Male, 28 years) (Code H5)

**Table 3:** Quotes reflective of the theme “Medication related challenges” and its sub-themes.

3.3. Main Theme Medication-Related Challenges	
3.3.1. Sub-theme-1: Adverse effects of injection	
“Yes, I had faced a lot of issues while taking the injections. I had severe pain, there were nodules formed at the injection site. I went through a terrible experience”	Patient (Female, 22 years) (Code B10)
“It was very painful to see her. I wonder how she used to take it every day as it was painful for her and even if I think about it, I used to be scared! The pain that she has undergone is unbearable. I used to cry just by looking at her injection site, and just imagine what she had gone through when she was getting it (injection) daily”	Caregiver (Female, 54 years, Mother) (Code B10F)
**3.3.2. Sub-theme-2: Adaptation challenges & adverse physical & psychological effects of pills**	
“Yes, there were a lot of challenges; the most difficult and frustrating thing was consuming the tablets. Whenever I used to consume tablets, I used to vomit; the health staff had told me to consume them even after vomiting and I used to follow the same. Completing the treatment is a challenging task…. It will be 400 to 600 mg and it is difficult to swallow” …	Patient (Female, 24 years) (Code B7)
“It was very difficult for my husband to take those tablets because of the very high-power medicines. In those two years, he became very weak because of the tablet dosage”	Caregiver (Mother, 54 years) (Code H10F)

**Table 4:** Quotes reflective of theme “lack of support and resources” and its sub-themes.

3.4. Main Theme-3: Lack of Support and Resources	
3.4.1. Sub-theme -1: Lack of social support and family distancing	
“I don’t know what to say. But in my mind, I was thinking about what has happened to me. What is there for me and why any of my friends are not coming to see me? And no one was calling me and asking how you are and what has happened to you and I use to cry at that time”	Patient (Male, 32 years) (Code H7)
“They came to the hospital. We told them about my condition. However, nobody helped us during our testing times. No one was bothered nor tried to help us in any way. It was very painful madam (emotional and started weeping). When I told my mother-in-law, she was least bothered”	Caregiver (Female, 47 years, Wife) (Code B9F)
**3.4.2. Sub-theme-2: Lack of resources**	
“I was not able to work. I stayed at home for three months. I was managing financially by borrowing money from my dad; I also have a debt to pay back. I have requested so many times and I have borrowed approximately. Rs.1 lakh”	A patient (Male, 33 years) (Code B8)
“my in-law family has helped at that time. Sometimes they will give rice and sometimes they will give money to buy something like eggs and mutton. With their help only we have survived for one year, for they have helped us one complete year”.	A patient (Male, 20 years) (Code H5)
**3.4.3. Sub-theme-3:** **Lack of care at the hospital**	
“No we did not face it. But what I think is, inviting the patients for the meetings and discussing the issues in-front of the crowd is kind of a demotivating them. Especially while conducting the survey. Once they called us for the survey saying: People who are infected with TB must come to the healthcare facility. But we did not like it, whatever it is, they are supposed to keep confidentiality and that was not the way right?”	Caregiver (Female, 23 years, living partner) (Code B5F)

## Data Availability

All relevant data are included in this publication. Audio files and transcripts of this study contains sensitive and personal information about patients and families and thus will not be shared to maintain participant confidentiality.

## Supplementary Materials

The following supporting information can be downloaded at: https://www.mdpi.com/article/10.3390/antibiotics11111586/s1, File S1: COREQ (COnsolidated criteria for REporting Qualitative research) Checklist; File S2: Quotes reflective of main themes and sub-themes.
