# Supplementary material for: A Dual Perspective of Psycho-Social Barriers and Challenges Experienced by Drug-Resistant TB Patients and Their Caregivers through the Course of Diagnosis and Treatment: Findings from a Qualitative Study in Bengaluru and Hyderabad Districts of South India
Source: Antibiotics (Basel). 2022 Nov 10;11(11):1586. doi: 10.3390/antibiotics11111586 (PMC9686660; doi:10.3390/antibiotics11111586)
Supplement: Supplementary file 1 [file antibiotics-11-01586-s001.zip › Supplementary File_S2.pdf]

Table S2. Quotes reflective of the theme “Emotional issues and social barriers” and its subthemes.

**3.2. Main Theme-1 Emotional Issues And Social Barriers**	
**3.2.1. Sub- theme-1: Fear and emotional distress due to disease status**	
“I was feeling sad and hurt for suffering from it (TB). I felt that why it has happened to such a good human being. I don’t have a habit of smoking Beedi, cigarettes then why me?”	*Patient (Male, 37 years). (Code B4)*
“My husband does not have any bad habits like smoking cigarettes and drinking alcohol, then why did this thing happen to him? By thinking of that I used to feel very upset. My heart was seriously hurt because of his illness, as he was not eating anything (chewing tobacco) or smoking or drinking	*Caregiver (Female, 24 years, Housewife).*<br>*(Code B4 F)*
“I was completely broken and thought that it is all over now and I will not get a chance to get into my mom’s hand again. I became so ugly that I was flat and thin and no dresses were fitting me. I completely lost my hope of survival, not interested in anything”	*Patient (Female, 25 years) (Code B3)*
“She used to tell me often that, Mom, I can’t, I can’t…. I cannot breathe mom; my breathing is going up and I am not able to and I will not be able to survive.”	*Caregiver (Female, 45 years, Mother & Housewife) (Code B3F)*
“I was not getting other thoughts. I usually become adamant and react oppositely, if the other person tries correcting me. For Example, I will follow this way only looking at the particular tablet; I will feel like vomiting and also will have that anxiety in mind. When the time to consume that particular medicine, I will have a mindset that I would vomit. With only one particular tablet I had difficulty swallowing. Mentally I had fixed my mind that if I take that (medicine) then I am going to vomit. So that is why I had trouble swallowing”.	*Patient (Male, 33 years) (Code B1)*
“He used to get angry with me and my daughters without reason. He was not like this before. His behaviour changed once he started to take the treatment. Now after his recovery, he is much better and getting back to the normal way. He used to get angry when he was sick and in treatment. If I tell him to eat food on time as I had to go to work, he used to get angry about that also. He used to respond by saying that “Just because you are going to work and earning money you are treating me like this”. Hey it is very difficult madam to take care of him”	*Caregiver (Female, 47 years) (Code B9F)*
“Vomiting and motion problems were there for him. By seeing his illness, I became very depressed and thought that after taking this many tablets also his disease is not getting cured”	*A patient caregiver (Female, 25 years, wife (Code H1F)*

3.2.4. Sub-theme- 4: Decisive moments of breakdown	
"When I asked the reason, he told, I can only survive up to only 40 years. Your validity is 40 or 45 plus because of your disease condition. I went there with higher hopes but he pulled me down with no hopes left inside. I lost my mind there only…. And soon after reaching home, I decided to quit my life"	*Patient (Male, 28 years) (Code B5)*
"At that time after taking the injection, there will be a lot of difficulties sir. For two months I have to take injections daily even though it was on Sundays or holidays. For ten days I took an injection and stopped one day, again they said the injection will not work if I stopped for a day. They told me that again I have to take back all the ten days of injection. At that time my health was very weak so I lost my hope".	*Patient (Male, 40 years) (Code H5)*

Table S3. Quotes reflective of theme "Medication related challenges and its subthemes.

3.3. Main Theme Medication-related challenges	
**3.3.1. Sub-theme- 1: Adverse effects of injection**	
"At that time, after taking the injection there will be a lot of difficulties sir. For two months I have to take an injection daily, whether it is Sundays or holidays, daily I have to take the injection. After taking the injection I was not able to go out of the house. In my first course, I used to go to work but in the second course I was not able to go out …. Pain will be there and I was not able to stand as the injection dose was high like 0.75ml. After taking the injection I was not able to eat even food"	*A patient (Male, 40 years) (Code H5)*
**3.3.2. Sub-theme 2: Adaptation challenges and adverse physical and psychological effects of pills**	
"Initially, I took medicines for 5 to 10 days on empty stomach. However, after taking the medicine I usually used to lose balance to even stand. I have fallen down so many times. For this, I went and compliant to madam about this"	*Patient, Female, 25 years, Sales girl in Pothys shopping mall) (Code H3)*

"Taking medicines was the hard part. No, I used to take it all. However, I was given 15 tablets at a time - making it a little difficult. Yes. It was about 750 mgs in total, and thinking about it makes me scared. The prescription consisted of tablets ranging from 100 mg and above. There were small ones and big ones. The latter were more in number. There were tablets of 500 mg and even 800 mg. I had to take 2-3 tablets as such! I took all the tablets at once. That is why the suffering was unbearable"	*Patient (Male, 56 years,) (Code B9)*
"I think the duration of the TB treatment is based on the stages of the TB. I don't know much about the stages and what goes next. In my opinion, is concerned the duration of TB must be reduced. For the patient, mentally, the duration matters. Few people think 9 months is a small duration, but for others, it is too much. I think the duration of the drug must be reduced by improving the quality of the medications"	*Caregiver (Female, 23 years, living partner) (Code B5F)*

Table S4. Quotes reflective of theme "lack of support and resources" and its subthemes

**3.4. Main Theme-3 : lack of support and resources**	
**3.4.1. Sub-theme -1: Lack of social support and family distancing**	
"No, I used to sit and stand on my own. I was not too much ill at that time. I was able to walk, but only thing is that I was not able to go to work. I started having a thought in my mind that I became a burden to my family. My wife and children are not with me because my parents have sent my wife and children to my mother-in-law's house. My parents told them that they should not stay with me until I recovered from the disease because my parents fear that the disease will be transmitted to my children	*A Patient (Male, 32 years) (Code H7).*
**3.4.2. Sub-theme 3 : Lack of resources**	
"I didn't give them (moneylender) the full amount, still I have to give. Now my sister and brother are helping us, however, I didn't inform my husband. At that time, I had to borrow, that is the only option I had. If I didn't have a timely meal, I can still survive, but I have to give my husband something to eat to fill his stomach. Sometimes my husband used to ask me where am I getting the money?. I used to tell him not to think about the money, I will manage it"	*Caregiver (Wife 30 years) (Code H5F)*
See mam to run my family I need Rs.6000 every month and if they give me Rs. 500 per month, there is no great difference, it was not enough. I cannot run my family with that little amount mam.	*Patient (Male, 37 years). (Code B4)*

No mam I did not go for work anywhere at that time. I used to get money as I am physically challenged and Rs. 500 from the XXX; this way I was managing the expenses of the house and took care of the family….They will give it for medicine and fruits it seems, but we didn't receive regularly. It was not that useful for us because I did not get the money consistently, I got it for only 3 or 4 months.	*Caregiver (Female, 25 years wife) B4F*
Hey that Rs. 500 how itgot spent I did not realize. If you go to buy vegetables, that 500 rupees will get over by a second. Where will it remain tell me (Laughing hysterically)…Yes madam. When you don't have single penny even ten ten paise is very precioius. Once my husband's bike got punctured and but there is no money for filling the puncture and there is no money to fill the petrol also, at that time Rs. 500 also is very helpful.	*Caregiver (Female, 47 years) (Code B9F)*
**3.4.3. Lack of care at the hospital**	
The staff (nurses, doctors and house-keeping staff) of the health facility, where I was admitted were behaving indifferently as though patient is going to bite them. Firstly, if we go to visit the doctor, they tell us to maintain distance from them. Whatever you have to discuss, kindly discuss maintaining distance; as you are suffering from TB, what is the need to come near me? Secondly, the food they serve is not good. Just for the sake of giving they will cook some random things and serve us. If we go to collect the food they treat us as untouchables. Thirdly, the cleaning staff don't maintain cleanliness.<br><br>*Even I agree that treatment is the best but the staff working there are not good they ignore patients and behave harshly with the patient.*	*Patient (Male, aged 33 years) (Code B1)*
